# Morton, Gould, and Bias: A Comment on “The Mismeasure of Science”

**DOI:** 10.1371/journal.pbio.1002444

**Published:** 2016-04-19

**Authors:** Michael Weisberg, Diane B. Paul

**Affiliations:** 1 Department of Philosophy, University of Pennsylvania, Philadelphia, Pennsylvania, United States of America; 2 Department of Political Science, University of Massachusetts Boston, Boston, Massachusetts, United States of America; Massey University, NEW ZEALAND

## Abstract

A closer look at Stephen Jay Gould’s criticisms of Samuel Morton vindicates Gould’s accusations of racial bias in Morton’s cranial measurements.

Stephen Jay Gould famously used the work of Samuel George Morton (1799–1851) to illustrate how unconscious racial bias could affect scientific measurement. Morton had published measurements of the average cranial capacities of different races, measurements that Gould reanalyzed in an article in *Science* [1] and then later in his widely read book *The Mismeasure of Man* [2]. During the course of this reanalysis, Gould discovered prima facie evidence of unconscious racial bias in Morton’s measurements. More than 30 years later, Lewis et al. published a critique of this analysis [3], denying that Morton’s measurements were biased by his racism. Instead, they claim that their “results falsify Gould’s hypothesis that Morton manipulated his data to conform with his a priori views.” We believe this is mistaken, and our comment will explain why.

Morton was a Philadelphia physician and highly respected scientist who avidly collected and measured human skulls. Between 1830, when he began his collection, and his death in 1849, Morton had amassed over a thousand specimens, making his the largest collection of human skulls in the world. To measure cranial capacity (a proxy for brain size), Morton filled the cranial cavities with spherical materials: “white pepper seed” for his 1839 measurements and BB shot for his 1844 measurements. He then computed racial averages using the 5-fold classification—Caucasian, Mongolian, Malayan, Ethiopian, and American—invented by Johann Friedrich Blumenbach (1752–1840). He also computed averages for families and subfamilies within these racial groups. His measurements indicated that the “Teutonic Family” (consisting primarily of Germans, Anglo-Saxons, Anglo-Americans, and Anglo-Irish) within the modern Caucasian group had by far the largest brains.

Gould’s argument for Morton’s unconscious racial bias is based on a comparison between two sets of measurements using two different materials. In his 1839 *Crania Americana*, Morton used “pepper seeds,” but he switched technique to using lead BB shot for the measurements presented in his later works, especially the 1844 *Crania Aegyptiaca*. Morton made this switch because the pepper seeds were light, variable in size, and easily compressed, and as a result his measurements were highly variable. It is important to note that Gould agrees with Morton about the superiority of the shot measurements. Gould calls these measurements “objective, accurate, and repeatable” [2].

After tabulating and reanalyzing Morton’s data, Gould was struck by a systematic difference between the two sets of measurements. The mean cranial capacity for Africans, Americans, and Caucasians had all increased between 1839 and 1844, as is shown in Fig 1. However, they did not change by the same amounts. The African skulls have a much larger increase in mean cranial capacity than the Americans and Caucasians. If this difference were the result either of lack of precision or of a systematic measurement error, the change should be approximately the same for the different races, but it was not. Gould thought that the best explanation for the more dramatic change in the African mean was unconscious manipulation on Morton’s part in 1839, when technique made that manipulation possible.

**Fig 1:**
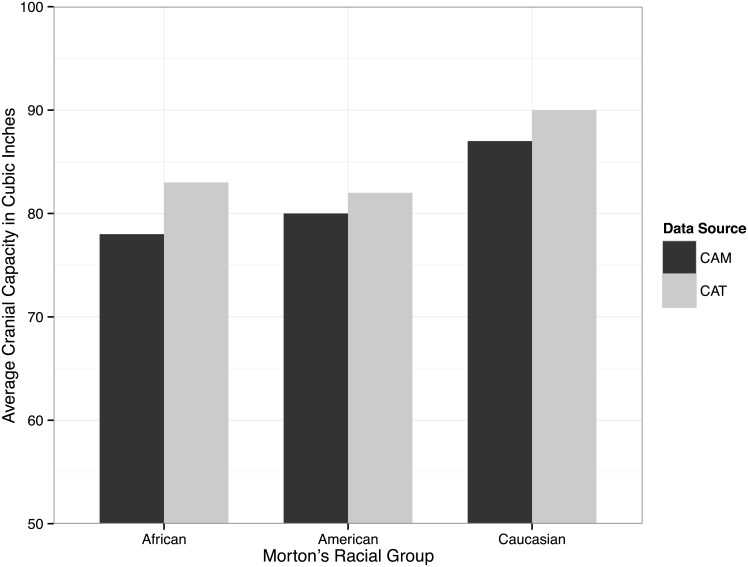
Change in mean cranial capacity from *Crania Americana* (CAM) to *Catalogue of Skulls of Man and the Inferior Animals* (CAT).

Why, then, do Lewis et al. think Morton has been vindicated? Their 2011 paper reports on the remeasurement of about half the skulls in Morton’s original set. They found that Morton’s shot measurements were mostly accurate, and that such errors as existed did not support a charge of bias. They also considered Gould’s other criticism of Morton’s methods and analysis, which they also judged to be mostly without merit (we are here only concerned with the measurement issue; for detailed discussions of all the claims in dispute, see [4,5]). They concluded that “Morton did not manipulate data to support his preconceptions, contra Gould” [3].

We take no issue with Lewis et al.’s remeasurements, but argue that these measurements are not and cannot be evidence for their conclusion. Although Lewis et al. found Morton’s shot-based measurements to be accurate, Gould already accepted this. Indeed, Gould had to assume that Morton’s shot measurements were accurate, as he relied on them in his own analysis. Gould never made, nor did he ever claim to make, nor did he have any reason to make any measurements himself. Gould’s argument depends on the difference between the two sets of measurements. Thus, as a matter of logic, there is no way that the results of Lewis et al.’s remeasurement program could be used to adjudicate the issue of who was biased. The many commentators who cite as a major failing of Gould’s that he “never bothered to measure the skulls himself” [6] have also, though perhaps more understandably, missed the point.

It is perfectly reasonable for a reader to have further questions about Morton’s measurements and samples before drawing a final verdict. Perhaps there are other explanations for the anomalously small African mean cranial capacity reported in *Crania Americana*. However, Lewis et al.’s remeasurements shed no light on this anomaly and only serve to highlight it further by demonstrating that Morton’s measurements with shot were indeed accurate. Gould’s claim that this is prima facia evidence of unconscious bias in *Crania Americana* remains intact.

Lewis et al. also allege that, according to Gould, studies of human variation are inevitably biased. Or, as is their view, “are objective accounts attainable, as Morton attempted?” But here, too, the critique misses its mark.

Gould argued that unconscious bias is ubiquitous in science. He actually praised Morton for the “rare and precious gift” of having published all his primary data, thus enabling others to check his work. Gould did not believe that biased results are inevitable. In his view, the tendency to fudge could and should be countered by “vigilance and scrutiny;” that is, by greater self-reflection and by cultivating, “as Morton did, the habit of presenting all our information and procedures, so that others can assess what we, in our blindness, cannot” [1]. In his view, only if we “understand and acknowledge inevitable preferences” can we countermand their influence [2]. Lewis et al. conclude that, contra Gould, “biased scientists are inevitable, biased results are not.” But this was precisely Gould’s own view!

Lewis et al. have charged that Gould’s “own analysis of Morton is likely the stronger example of bias influencing results” [3]. We maintain that this accusation, which continues to reverberate, is undeserved, and we hope that this Comment will prompt at least some readers to reevaluate the evidence and arguments.

**Box 1. Editor’s Note**

*PLOS Biology* invited the authors of [3] to respond to this Formal Comment. They provided the following statement: “We decline to respond as the issues raised are well covered in our original paper, which we encourage interested readers to consult.”
